# iDNA-OpenPrompt: OpenPrompt learning model for identifying DNA methylation

**DOI:** 10.3389/fgene.2024.1377285

**Published:** 2024-04-16

**Authors:** Xia Yu, Jia Ren, Haixia Long, Rao Zeng, Guoqiang Zhang, Anas Bilal, Yani Cui

**Affiliations:** ^1^ School of Information and Communication Engineering, Hainan University, Haikou, Hainan, China; ^2^ School of Information Science and Technology, Hainan Normal University, Haikou, Hainan, China

**Keywords:** DNA methylation, OpenPrompt learning, prompt template, prompt verbalizer, BERT tokenizer

## Abstract

**Introduction:** DNA methylation is a critical epigenetic modification involving the addition of a methyl group to the DNA molecule, playing a key role in regulating gene expression without changing the DNA sequence. The main difficulty in identifying DNA methylation sites lies in the subtle and complex nature of methylation patterns, which may vary across different tissues, developmental stages, and environmental conditions. Traditional methods for methylation site identification, such as bisulfite sequencing, are typically labor-intensive, costly, and require large amounts of DNA, hindering high-throughput analysis. Moreover, these methods may not always provide the resolution needed to detect methylation at specific sites, especially in genomic regions that are rich in repetitive sequences or have low levels of methylation. Furthermore, current deep learning approaches generally lack sufficient accuracy.

**Methods:** This study introduces the iDNA-OpenPrompt model, leveraging the novel OpenPrompt learning framework. The model combines a prompt template, prompt verbalizer, and Pre-trained Language Model (PLM) to construct the prompt-learning framework for DNA methylation sequences. Moreover, a DNA vocabulary library, BERT tokenizer, and specific label words are also introduced into the model to enable accurate identification of DNA methylation sites.

**Results and Discussion:** An extensive analysis is conducted to evaluate the predictive, reliability, and consistency capabilities of the iDNA-OpenPrompt model. The experimental outcomes, covering 17 benchmark datasets that include various species and three DNA methylation modifications (4mC, 5hmC, 6mA), consistently indicate that our model surpasses outstanding performance and robustness approaches.

## 1 Introduction

DNA methylation is essential for numerous biological processes and is associated with multiple diseases, particularly cancer (Maegawa et al., 2010; Yehudit and Howard, 2013). Accurately identifying DNA methylation sites is necessary for comprehending gene regulation and the mechanisms of diseases. Deep learning approaches have recently emerged as a significant tool in recognizing DNA methylation sites, demonstrating encouraging outcomes. Presently, three extensively studied DNA methylation types include N6-methyladenine (6mA), 5-hydroxymethylcytosine (5hmC), and N4-methylcytosine (4mC) (Manavalan et al., 2019; Yingying et al., 2021).

The field has recently witnessed notable advancements in integrating deep learning methodologies. Regarding the prediction of DNA methylation sites of 4-mC species, in 2019, introducing two remarkable algorithms, 4mCCNN (Khanal et al., 2019) and 4mCPred-SVM (Leyi et al., 2019), marked a leap in 4-mC prediction capabilities. 4mCCNN used a CNN-based framework, whereas 4mCPred-SVM was developed using support vector machine (SVM) techniques. Additionally, Quanzhong et al. (2020) crafted DeepTorrent, a composite model fusing CNN and BiLSTM, to identify 4-mC sites (Quanzhong et al., 2020). Deep4mC, another innovative algorithm, validated the effectiveness of a CNN-only approach in delivering impressive 4-mC prediction outcomes (Haodong et al., 2020). Hyb4mC introduced a unique approach, integrating an elastic net with a capsule network for smaller datasets while emphasizing the prowess of CNN for larger datasets (Ying et al., 2022). Moreover, Zeng et al. introduced a novel two-layer deep learning structure named Deep4mcPred, based on ResNet with long short-term memory (LSTM) (Rao and Minghong, 2020). Xia et al. (2023) presented the DRSN4mCPred model, a variant based on the deep residual network, and it can enhance the model’s capability to assimilate intricate data characteristics (Xia et al., 2023).

The research focusing specifically on recognizing 5hmC sites is comparatively limited. Tran TA et al. applied a unique feature extraction approach using k-mer embeddings obtained from a pre-trained language model (Duong et al., 2021). The BiLSTM-5mC model leveraged one-hot encoding and nucleotide property and frequency (NPF) techniques for representing nucleotide sequences. It then integrated a bidirectional long short-term memory (BiLSTM) model with a fully connected network to forecast methylation sites (Xin et al., 2021).

The field has seen considerable research in identifying 6-mA methylation sites. For instance, the sNNRice6mA algorithm adopted a two-dimensional one-hot encoding approach for DNA sequences, using a convolutional neural network (CNN) to identify 6-mA sites (Haitao and Zhiming, 2019). Ying et al. (2021) incorporated an attention mechanism into their model, enhancing the identification of critical features for more accurate detection of epigenetic changes in DNA (Ying et al., 2021). Mehedi et al. (2020) developed Meta-i6mA, a cross-species predictive framework for 6-mA sites in plant genomes, leveraging informative features in a comprehensive machine learning methodology (Mehedi et al., 2020). Juntao et al. (2021) introduced DeepM6ASeq-EL, an advanced method combining LSTM with ensemble learning to predict human m6A sites in RNA with high accuracy (Juntao et al., 2021). This fusion of techniques significantly boosts the model’s prediction accuracy, offering a powerful tool for m6A site identification in the human genome. Sho et al. (2022) used word to vector (word2vec) and Bidirectional Encoder Representations from Transformers (BERT) for developing BERT6mA, a deep learning framework that showed exceptional performance in predicting 6-mA modifications (Sho et al., 2022). Ue et al. (2022) proposed a CapsuleNet-based DNA m6A site recognition framework, proving its precision in methylation site prediction (Ur et al., 2022). Sho et al. (2022) demonstrated that BERT-based models could significantly enhance the accuracy of predicting 6-mA sites in DNA, effectively handling interspecies variations and serving as a valuable asset for plant genome studies and epigenetic research (Sho et al., 2022).

Although the methods mentioned earlier have achieved varying degrees of progress, they are all specifically designed to identify one type of DNA methylation. Conversely, there are only a few techniques that address all three previously mentioned methylation categories (Lv et al., 2020; Yingying et al., 2021; Junru et al., 2022), with notable examples being iDNA-ABT (Yingying et al., 2021), iDNA-ABF (Junru et al., 2022), and iDNA-MS (Lv et al., 2020). Typically, DNA methylation datasets appropriate for deep learning contain shorter sequences per sample, with sequences of 41 base pairs (bp) being predominantly prevalent.

Many studies indicate a growing interest in using deep learning to predict DNA methylation, achieving significant progress in enhancing prediction accuracy (Wang et al., 2023). However, current deep learning-based models have not completely exploited the capabilities of learning features. Acknowledging this gap, the genomic sequences can be viewed as biological texts, and the sequences’ bases can be considered biological words (Zou et al., 2019; Dai et al., 2022). Considering this, we propose the iDNA-OpenPrompt model, an OpenPrompt learning approach (Ding et al., 2021) for DNA methylation sequences. The model combines a prompt template, prompt verbalizer, and pre-trained language model (PLM) to construct a prompt learning framework.

Moreover, a DNA vocabulary library, BERT tokenizer, and specific label words are also introduced into the model to enable accurate identification of DNA methylation sites. An extensive analysis is conducted to evaluate the predictive performance, reliability, and consistency of the iDNA-OpenPrompt model. The results, which include 17 benchmark datasets covering a variety of species and three types of DNA methylation modifications (4 mC, 5 hmC, and 6 mA), consistently reveal that our model surpasses other outstanding methods in both performance metrics and overall robustness.

The primary contribution of this article is that the iDNA-OpenPrompt model can learn biological contextual semantics. In contrast to the existing approaches, iDNA-OpenPrompt brings the following contributions:

(1) Our model creates a DNA vocabulary library and integrates it with the BERT tokenizer for DNA methylation sequences to develop the prompt template.
(2) Our model constructs label words specific to DNA methylation sequences and integrates them with the BERT tokenizer to establish a prompt verbalizer.
(3) Our model constructs an OpenPrompt learning model that can be used for identifying DNA methylation sites.

## 2 Materials and methods

### 2.1 Dataset

For the iDNA-OpenPrompt model’s evaluation, the datasets are selected from the iDNA-MS web server (iDNA-MS, 2020), including training and independent testing subsets, as detailed in Table 1. There are 4mC, 5hmC, 6mA methylation sequences, totaling 17 datasets, encompassing 501,200 DNA sequences. The length of each sample in the datasets is 41 base pairs. It is worth mentioning that in the 6mA samples, the methylated adenine (A) is always found in the central position, and similarly, methylated cytosine (C) is prominent in the 5hmC and 4mC samples. Indeed, such central position characteristics are also present in the negative samples.

**TABLE 1:** Overview of datasets.

ID	Dataset	Training		Independent testing	
		Positive	Negative	Positive	Negative
1	4mC_C.equisetifolia	183	183	183	183
2	4mC_F.vesca	7,899	7,899	7,898	7,898
3	4mC_S.cerevisiae	990	990	989	989
4	4mC_Tolypocladium	7,664	7,664	7,663	7,663
5	5hmC_H.sapiens	1,172	1,172	1,172	1,172
6	5hmC_M.musculus	1840	1840	1839	1839
7	6mA_A.thaliana	15,937	15,937	15,936	15,936
8	6mA_C.elegans	3,981	3,981	3,980	3,980
9	6mA_C.equisetifclia	3,033	3,033	3,033	3,033
10	6mA_D.melanogaster	5,596	5,596	5,595	5,595
11	6mA_F.vesca	1,551	1,551	1,551	1,551
12	6mA_H.sapiens	9,168	9,168	9,167	9,167
13	6mA_R.chinensis	300	300	300	300
14	6mA_S.cerevisiae	1893	1893	1893	1893
15	6mA_T.thermophile	53,800	53,800	53,800	53,800
16	6mA_Tolypocladium	1,690	1,690	1,689	1,689
17	6mA_Xoc BLS256	8,608	8,608	8,607	8,607

Table 1 includes a “dataset” column, which lists the names of the various datasets. Within these names, the part before the “-” separator signifies the methylation modification type, and the segment following the separator denotes the species type. The “training” and “testing” columns provide detailed information about the quantity of positive and negative samples within each dataset.

### 2.2 Overview of iDNA-OpenPrompt

**FIGURE 1:**
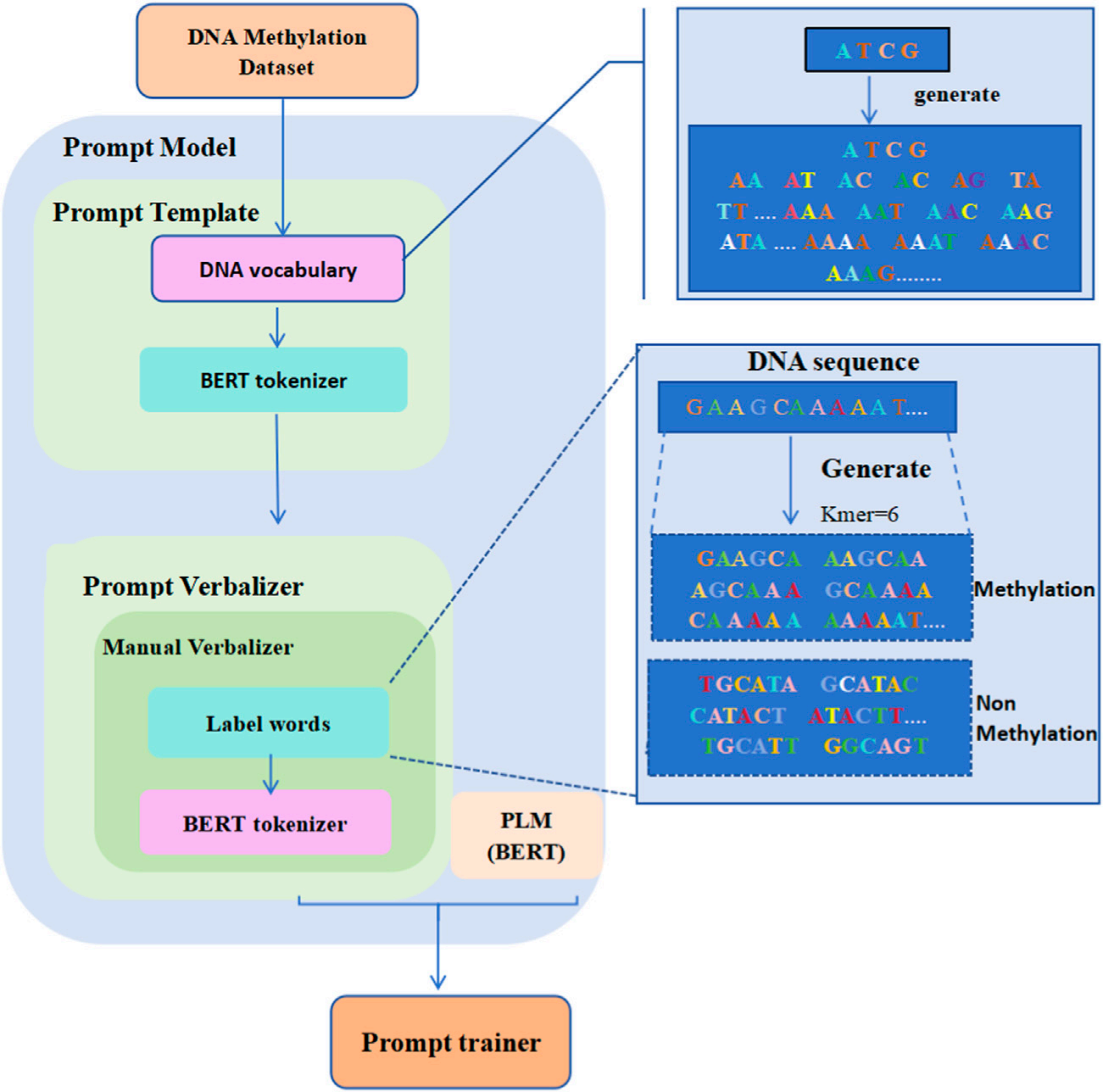
Overall architecture of the iDNA-OpenPrompt Model.

Figure 1 displays the overall structure of the iDNA-OpenPrompt model. The core module of the iDNA-OpenPrompt model (prompt model) mainly consists of three parts: the prompt template, prompt verbalizer, and PLM. The prompt template part involves building a DNA vocabulary library and training it in the transformer’s BERT tokenizer to form the prompt template. In the prompt verbalizer part, label words for DNA methylation sequences are created, and the constructed label words, along with the transformer’s BERT tokenizer, are used to build a prompt verbalizer in the manual verbalizer method of OpenPrompt learning. The BERT model, which can capture bidirectional contextual information in the text, is used for the PLM part. Below, the key technologies of the iDNA-OpenPrompt model will be introduced.

### 2.3 Prompt learning

In a standard prompt learning setting, like in natural language processing (NLP) tasks, input sentences are structured through a natural language template. This process frames text classification tasks as cloze-style tasks (Zhu et al., 2023). For example, in a task of classification, the goal is to categorize the sentence x into various topics, such as “I must reduce the budget” into the label. $y_{1}=BUSINESS$ or $y_{2}=SPORTS$ , and the template could be expressed as Eq. (1):

$$x_{p}=\left[CLS\right]x,a\left[MASK\right]question.$$ (1)

Given an input $x=\left\{x_{1},x_{2},\cdots ,x_{n}\right\}$ , categorized into a label y from the set of labels Y, the corresponding label word set is represented as $V_{y}=\left\{y_{1},y_{2},{\cdots ,y}_{n}\;\right\}$ . Here, $V_{y}$ is a subset of the vocabulary V and associated with the y category. In PLMs, denoted as P, the probability of each word v in $V_{y}$ being used to fill in the [MASK] is represented by $p\left(\left[\text{MASK}\right]=v\in V_{y}|x_{p}\right)$ . As a result, the text classification task is reformulated by calculating the probabilities of label words. This computation is formulated as Eq. (2):

$$p\left(\left\{y\in Y|x\right\}\right)=p\left(\left[\text{MASK}\right]=v\in V_{y}|x_{p}\right).$$ (2)

In this example, if the determined probability for $V_{1}=\left\{business\right\}$ , corresponding to $y_{1}=BUSINESS$ , exceeds that of $V_{2}=\left\{sports\right\}$ for $y_{2}=SPORTS$ , it suggests that the sentence x belongs to the BUSINESS category.

### 2.4 OpenPrompt

OpenPrompt (Ding et al., 2021) is an open-source toolkit designed for prompt learning, offering both ease of use and extensibility. It effectively modularizes the entire prompt learning framework and considers the interactions between various modules. OpenPrompt enables the versatile integration of different task formats, PLMs, and prompting modules. An instance of this flexibility is the straightforward adaptation of prefix-tuning (Li and Liang, 2021) for text classification tasks within OpenPrompt. This capability allows users to evaluate the broad applicability of their prompt learning models across different tasks rather than just focusing on performance in specific tasks.

In OpenPrompt, the template class is specifically used to create or define textual or soft-encoding templates encapsulating the original input. The templates are pivotal in constructing and formatting input data for effective interaction with PLMs (Han et al., 2021). They can wrap original text data into a format that aligns with the structure of PLMs. Templates can add extra contextual information to aid the model in more effectively comprehending and handling the input data. The verbalizer bridges PLMs and specific task requirements, offering a flexible and effective way to customize model outputs.

### 2.5 Prompt template

The prompt template is to construct a prompt framework, which involves formatting the original input data (such as sentences or paragraphs) into a specific structure, making it more suitable for understanding and processing by PLMs. One or more mask tokens are often inserted (for example, the [MASK] token used in BERT).

Various studies have explored different types of templates. For instance, there are manually written templates (Schick and Schütze, 2020) and purely soft templates (Lester et al., 2021). Liu et al. (2023) demonstrated effective results by keeping manual tokens unchanged while fine-tuning a smaller portion (Liu et al., 2023). Han et al. (2022) used contextualized templates, necessitating the addition of specific entities to create complete templates. Additionally, their approach to loss calculation involved using outputs from various positions (Han et al., 2022). Logan IV et al. (2021) introduced an empty template, a straightforward combination of the input data, and a subsequent [MASK] token (Logan IV et al., 2021).

Within the iDNA-OpenPrompt model, the manual template, which is trainable using task-specific datasets, is used. This manual template enables the precise construction of templates based on one’s understanding of the task and specific requirements, and it can simplify the model training process and reduce the demand for computational resources. The template mainly consists of two modules: creating a DNA vocabulary library and the BERT tokenizer.

#### 2.5.1 Creation of the DNA vocabulary

When creating a vocabulary library for DNA methylation sequences, unlike in traditional NLP tasks, the presence of one, two, or even three nucleobases in a sequence does not necessarily indicate a DNA methylation site. Considering the categories of DNA methylation (4 mC, 5 hmC, and 6 mA) and the nucleobase composition for each, we propose using DNA vocabulary for DNA methylation sequences in the prompt template. Here, the length of nucleobase sequences (A, T, G, and C) is defined as kmer = 1, 2, 3, 4, 5, and 6, to form the DNA methylation sequence vocabulary. For example, at kmer = 1, the template includes four nucleobase words: A, T, G, and C. At kmer = 2, there are 16 nucleobase words, such as AA, AT, AG, … , and CC. Similarly, for kmer = 3, there are 64 nucleobase words; for kmer = 4, there are 256 nucleobase words; for kmer = 5, there are 1,024 nucleobase words; and for kmer = 6, there are 4,096 nucleobase words. The maximum k-mer value in this prompt template is set to 6 because, in DNA methylation sequences, 6 mA methylation involves attaching a methyl group to the sixth nitrogen atom of the adenine nucleobase. Therefore, the DNA vocabulary library contains a total of 5,460 nucleobase words. After creating the vocabulary library, the BERT tokenizer is used to generate the tokenizer of the iDNA-OpenPrompt model.

#### 2.5.2 BERT tokenizer

BERT tokenizer is designed explicitly for the BERT model and is pivotal in NLP tasks. The DNA vocabulary processed by the BERT tokenizer enables the raw text to be transformed into a format effectively handled by OpenPrompt learning. It breaks down basic text strings into smaller units, tokens, words, subwords, or symbols. To accommodate the needs of the BERT model, the BERT tokenizer automatically adds unique tokens such as the start of the sequence token [CLS], separator token [SEP], and padding token [PAD]. It creates an attention mask to indicate which tokens are meaningful and which are for padding. The BERT tokenizer provides essential text processing capabilities for the use of the iDNA-OpenPrompt model.

### 2.6 Prompt verbalizer

In OpenPrompt, the verbalizer plays an important role, especially when applying PLMs to downstream tasks. The primary function of the verbalizer is to map labels to the vocabulary; the verbalizer maps task-specific labels (such as category labels in classification tasks) to words within the pre-trained model’s vocabulary. This mapping allows the model to associate its outputs with specific labels.

Like prompt templates, prompt verbalizer classes derive from a shared base class featuring necessary attributes and essential abstract methods. Beyond the manually defined verbalizer, OpenPrompt includes automated options like the automatic verbalizer and knowledgeable verbalizer (Hu et al., 2021). Critical processes such as calibrations (Zhao et al., 2021) are also incorporated in OpenPrompt. In the iDNA-OpenPrompt model, a manual verbalizer is chosen for the prompt verbalizer; the manual verbalizer mainly consists of two modules: label words and BERT tokenizer.

#### 2.6.1 Label words

Labeling words is a crucial attribute in the manual verbalizer component within the OpenPrompt framework. These words or phrases are labeled words to interpret and transform the model’s output.

In this study, the method for constructing label words is as follows: for DNA methylation sequences and non-methylation sequences, centering around the 21st nucleobase of the sequences, kmer = 6 encoding is performed on the nucleobase sequences on both sides of the central nucleobase and the encoded words as label words. In all 4-mC sequences (including positive and negative samples), the 21st nucleobase is always C; in all 5-hmC sequences, it is C, and in all 6-mA sequences, it is A.

The words encoded from the positive samples in the DNA methylation sequence dataset are used as positive-sample label words. In contrast, those encoded from the negative samples are used as negative-sample label words.

**FIGURE 2:**
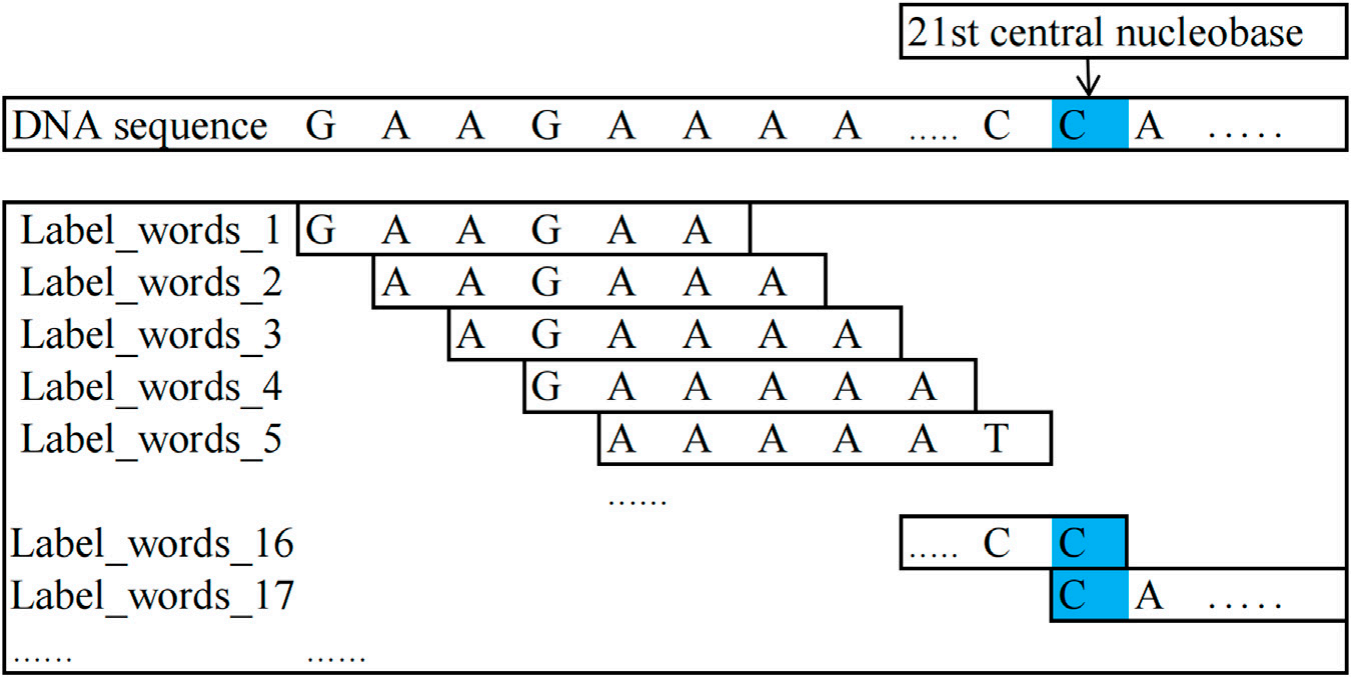
Schematic diagram of label_words for DNA methylation sequences.

For example, it is taking a positive sample from the 4-mC category of the 4 mC_F.vesca species, “GAA​GCA​AAA​ATC​GGA​AAA​CCC​A … CTTTTGGTT”: the possible positive sample label words that can be constructed are as follows: “GAAGCA, AAGCAA, AGCAAA, GCAAAA, … , AAAACC, AGAAAA, GAAAAT, AAAATT, … , TTGGTT”. Similarly, a negative sample was taken from the 4-mC category of the 4 mC_F.vesca species, “TGC​ATA​CTT​TCA​GTA​GTT​TTC​AAT … ATGGCAGT”: the negative sample label words that can be constructed are as follows: “TGCATA, GCATAC, CATACT, ATACTT, … , AGTTTT, AATGCA, ATGCAT, TGCATT, … , GGCAGT”. To understand the process of constructing label_words for DNA methylation sequences, Figure 2 illustrates its schematic diagram.

### 2.7 PLM

The PLM of iDNA-OpenPrompt is the BERT model. The application of the BERT model in OpenPrompt follows the fundamental principles and structure of the BERT model (Devlin et al., 2018) while adapting and extending it within the framework of prompt learning. The core of the BERT model is the encoder part of the transformer, which comprises multiple encoder layers, each containing self-attention mechanisms and feed-forward neural networks. One of the primary attributes of BERT is its ability to generate bidirectional contextualized word embeddings, signifying that it considers the context of the entire sentence when processing each word. To learn deep language representations, the BERT model undergoes pre-training on an extensive corpus, including tasks like the masked language model (MLM) and next sentence prediction (NSP).

#### 2.7.1 Attention calculation

The scalar product between the query vector (Q) and key vector (K) is computed, followed by scaling down of the result to prevent overly large attention scores, while a scaling factor (commonly the inverse square root of the key vectors’ dimension) is also factored in. The attention scores are then subjected to a softmax operation for normalization into attention weights. A weighted sum over the value vectors (V) is then performed using these weights, resulting in the final attention representation. The formulaic representation of self-attention is expressed as Eq. (3) and (4):

$$\left\{\begin{array}{c} Q=XW^{Q}\\ K=XW^{K},\\ V=XW^{V} \end{array}\right.$$ (3)

$$\text{Self}-\text{attention}\left(Q,K,V\right)=\text{softmax}\left(\frac{QK^{T}}{\sqrt{d_{k}}}\right)V.$$ (4)

In this context, $X\in R^{L*d_{m}}$ symbolizes the embedding output obtained from the embedding module, where $d_{m}$ indicates the embedding dimension and L represents the input sequence’s length. Q, K, and $V\in R^{L\times d_{k}}$ correspond to the matrices of the query, key, and value, respectively. These matrices are derived from X through a linear transformation using $W^{Q}$ , $W^{K}$ , and $W^{V}$ , each existing in the real space $R^{d_{m}*d_{k}}$ . Here, $d_{k}$ denotes the size of the query, key, and value vectors. $d_{m}$ and $d_{k}$ are both regarded as hyperparameters.

#### 2.7.2 Multi-head attention

The computation of the attention head specified by index “i” is as shown in Eq. (5), (6) and (7):

$$Q_{i}=XW_{i}^{Q},K_{i}=XW_{i}^{K},V_{i}=XW_{i}^{V},i=1,\cdots ,h,$$ (5)

$${Head}_{i}=Self-attention\left(Q_{i},K_{i},V_{i}\right)$$ (6)

$$\text{MultiHead}-\text{Attention}\left(Q,K,V\right)=\text{Concact}\left({\text{Head}}_{1},{\text{Head}}_{2},\cdots ,{\text{Head}}_{h}\right)W^{O}.$$ (7)

$W_{i}^{Q}$ , $W_{i}^{K}$ , and $W_{i}^{V}\in R^{d_{m}\times d_{k}}$ are the query, key, and value matrices for the i-th head, respectively. The parameter ‘h’ denotes the count of heads. The multi-head attention is used for Q, K, and V by concatenating ‘h’ individual heads, with each performing self-attention relevant to the input sequence. Furthermore, ${W^{o}\in R}^{d_{m}\times d_{k}}$ acts as a linear transformation matrix, adjusting the dimensions of the multi-head attention’s output to align with the input dimensions of the encoder block. This enables a skip connection, where the input for the encoder block is linked to the output from the multi-head attention mechanism.

In OpenPrompt, the BERT model is commonly used with templates and verbalizers. Prompt templates are designed to construct input formats suitable for processing by BERT. In contrast, prompt verbalizers are used to map the output of models to specific task labels by leveraging the advanced language understanding capabilities of the BERT model, which can strengthen the function of OpenPrompt models within a variety of NLP tasks.

## 3 Performance metrics

The performance of the iDNA-OpenPrompt model, along with other DNA methylation recognition models (Zeng and Liao, 2021; Li F. et al., 2023; Li Q. et al., 2023), is evaluated using the following five commonly used metrics: accuracy (ACC), sensitivity (SN), specificity (SP), Matthews’ correlation coefficient (MCC), and area under curve (AUC). The equations for these measurements are expressed below Eq. 8 to Eq. 12:

$$\text{ACC}=\frac{\text{TP}+\text{TN}}{\text{TP}+\text{FN}+\text{TN}+\text{FP}},$$ (8)

$$\text{SN}=\frac{\text{TP}}{\text{TP}+\text{FN}},$$ (9)

$$\text{SP}=\frac{\text{TN}}{\text{TN}+\text{FP}},$$ (10)

$$\text{MCC}=\frac{\text{TP}\times \text{TN}-\text{FP}\times \text{FN}}{\sqrt{\left(\text{TP}+\text{FN}\right)\left(\text{TP}+\text{FP}\right)\left(\text{TN}+\text{FP}\right)\left(\text{TN}+\text{FN}\right)}},$$ (11)

$$\text{AUC}=\frac{\sum_{i\in \text{pos}}{\text{rank}}_{i}-\frac{{\text{num}}_{\text{pos}}\left({\text{num}}_{\text{pos}}+1\right)}{2}}{{\text{num}}_{\text{pos}}{\text{num}}_{\text{neg}}}.$$ (12)

Here, TP, FN, TN, and FP denote the counts of true positive, false negative, true negative, and false positive instances, respectively. ACC and MCC are both used for gauging the model’s comprehensive performance. SN pertains to the ratio of accurately predicted samples correctly identified as methylated with the predictor, while SP quantifies the proportion of accurately predicted non-methylated samples with the predictor. The AUC is determined as the region enclosed between the receiver operating characteristic (ROC) curve and the coordinate plane, where the false positive rate (FPR) is plotted on the *x*-axis, and the true positive rate (TPR) is plotted on the *y*-axis. In total, an increase in these metrics signifies an improved model performance.

## 4 Results

### 4.1 The visualization of UMAP for samples of iDNA-OpenPrompt

To visually demonstrate the iDNA-OpenPrompt’s performance, Uniform Manifold Approximation and Projection (UMAP) (Junru et al., 2022) displays the distribution of samples with and without methylation sites. UMAP is a sophisticated non-linear method for reducing dimensionality that effectively maps high-dimensional data into a more manageable two-dimensional space, preserving local and global data point structures.

**FIGURE 3:**
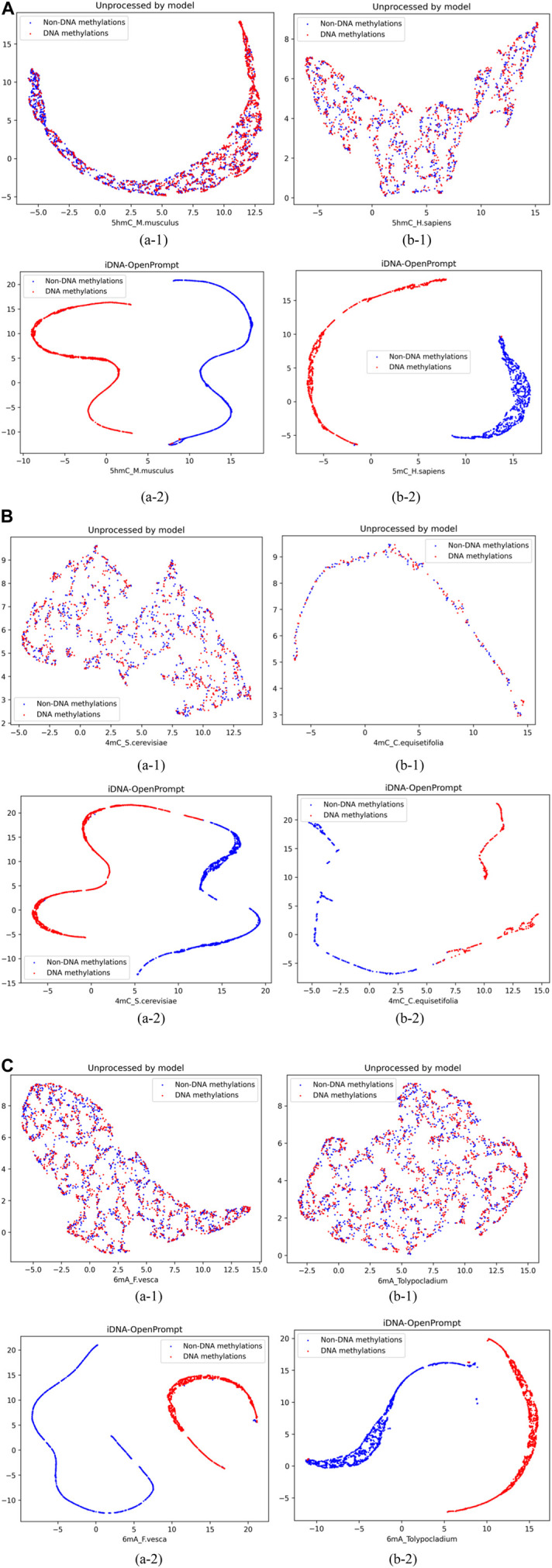
Representing samples before and after using the iDNA-OpenPrompt model with UMAP. **(A)** UMAP visualization of samples before and after processing with the iDNA-OpenPrompt model for the species 5hmC_M.musculus and 5hmC_H.sapiens. **(B)** UMAP visualization of samples before and after processing with the iDNA-OpenPrompt model for the species 4mC_cerevisiae and 4mC_C.equisetifolia. **(C)** UMAP visualization of samples before and after processing with the iDNA-OpenPrompt model for the species 6mA_F.vesca and 6mA_Tolypocladium. In Panels (A–C) (a-1) and (b-1) show the samples before processing with the model, while (a-2) and (b-2) show the samples after processing with the model.

As seen in Figure 3, blue corresponds to non-DNA methylation (negatives), while red corresponds to DNA methylation (positives). The figures of (a-1) and (b-1) display the visualization of DNA methylation and non-methylation sequence samples without model processing; positive and negative samples appear mixed. The figures of (a-2) and (b-2) exhibit the visualization of DNA methylation and non-methylation sequence samples after iDNA-OpenPrompt model processing; and the positive and negative samples distinctly separate into well-defined groups. This separation visually confirms the model’s capacity to differentiate between DNA methylation and non-DNA methylation samples effectively.

### 4.2 Comparison of iDNA-OpenPrompt’s performance with other outstanding methods

**FIGURE 4:**
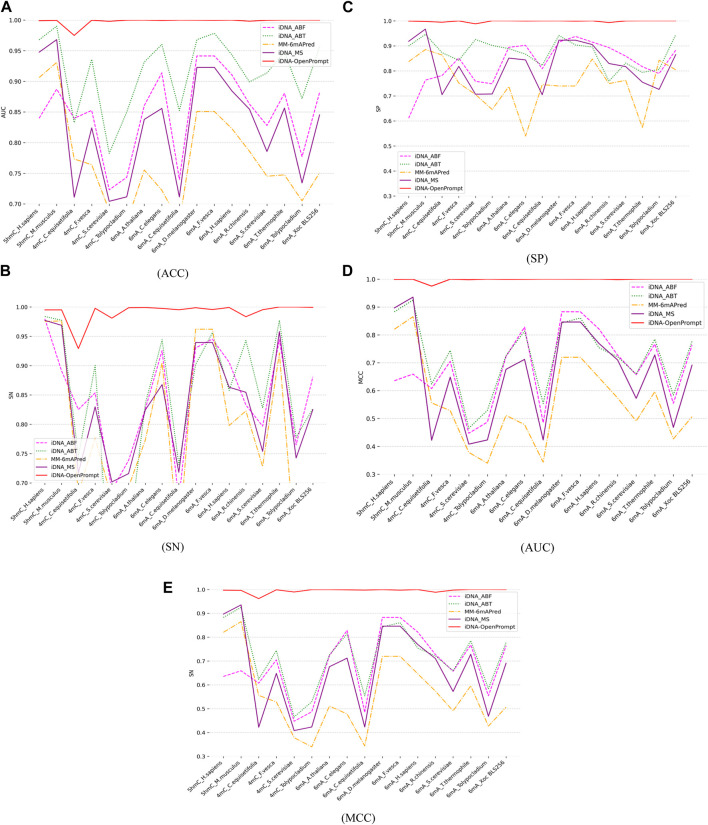
Comparing Performance of iDNA-OpenPrompt with other outstanding methods. **(A)** the ACC of iDNA-OpenPrompt with other outstanding methods, **(B)** the SN of iDNA-OpenPrompt with other outstanding methods, **(C)** the SP of iDNA-OpenPrompt with other outstanding methods, **(D)** the AUC of iDNA-OpenPrompt with other outstanding methods, **(E)** the MCC of iDNA-OpenPrompt with other outstanding methods. The evaluation metrics displayed above (ACC, SN, SP, AUC, MCC) are the results of testing the iDNA-OpenPrompt, iDNA-ABT, iDNA-ABF, iDNA-MS, and MM-6mAPred models on datasets of 17 species.

To evaluate the performance of iDNA-OpenPrompt, the comparative study is conducted against four outstanding predictors, including iDNA-ABT (Yingying et al., 2021), iDNA-ABF (Junru et al., 2022), iDNA-MS (Lv et al., 2020), and MM-6mAPred (Pian et al., 2020). iDNA-ABT, iDNA-ABF, and iDNA-MS are designed for various methylation prediction tasks, whereas MM-6mAPred was initially tailored for 6-mA site prediction. This comparison highlights iDNA-OpenPrompt’s adaptability and its capability, not just limited to 6 mA but also extending to 5hmC and 4 mC. Each of these predictors is independently trained on 17 distinct training datasets encompassing three methylation types, and then, its corresponding test dataset is evaluated (details are provided in Table 1). The outcomes, encompassing metrics such as ACC, SN, SP, AUC, and MCC, are depicted in Figure 4A–E. The data clearly show that the proposed model consistently surpasses the performance of four other exceptional predictors across all 17 datasets. The effectiveness of the proposed model can be attributed to its utilization of the OpenPrompt learning framework, which has proven to be highly effective in enhancing its performance, along with the outstanding performance of the prompt template and prompt verbalizer specifically designed for DNA methylation sequences.

### 4.3 Successful cross-species validation results

**FIGURE 5:**
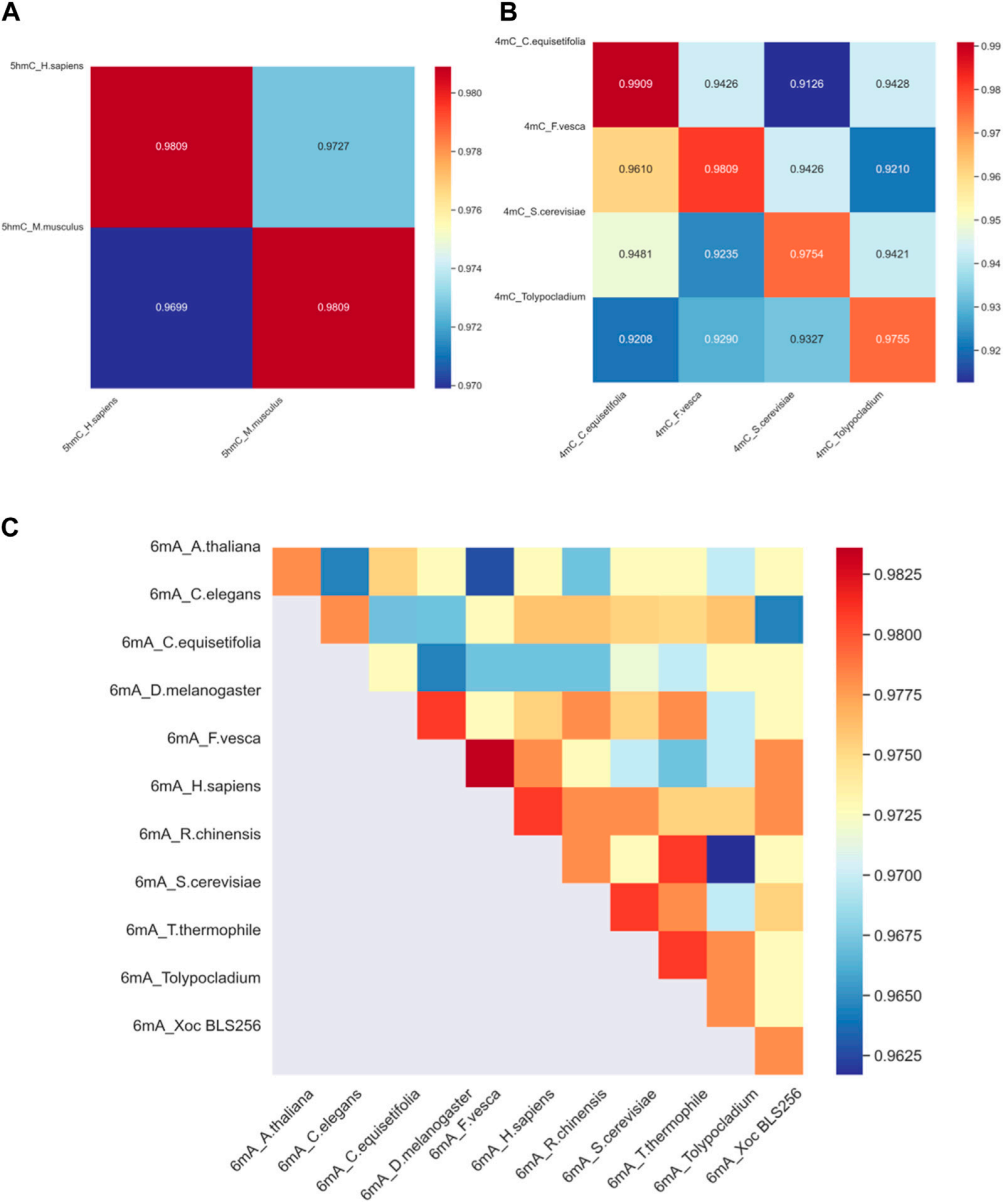
The heat map of cross-validation. **(A)** The cross-validation accuracy results for DNA methylation 5hmC in two species. **(B)** The cross-validation accuracy results for DNA methylation 4mC in four species. **(C)** The cross-validation accuracy results for DNA methylation 6mA in eleven species. In the figures, the species datasets indicated on the horizontal axis are used for training, and the species datasets indicated on the vertical axis are used for testing.

To assess the proposed model’s adaptability across different species, it is imperative to gauge a model’s ability to be trained on data from one species and then used to detect modification sites in others. With this goal in mind, we have developed distinct models, each customized for a specific species; the effectiveness of these models is ascertained by applying them to other species for 4mC, 5hmC, 6mA modification. The outcomes of this validation procedure across different species are visually represented in Figure 5.

Considering the significant discrepancy in the quantity of training and testing samples for various species, with some species having only a few hundred samples and others reaching over a hundred thousand, we aim for fairness in cross-validation. Therefore, from the datasets of all species, we randomly selected 365 samples for the model’s cross-validation. This selection comprised 183 positive samples and 182 negative samples. The cross-validation outcomes are depicted in Figure 5.

Figure 5A reveals the results of cross-species validation of 5hmC_*H. sapiens* and 5hmC_*M. musculus*. Specifically, the accuracy rate attained for 5hmC_H: sapiens and 5hmC_*M. musculus* is 98.09%, underscoring the success of the proposed method. Figure 5C reveals that in the 6mA_R.chinensis model’s cross-validation, the accuracy for 6mA_R.chinensis is less than that for 6mA_T.thermophile indicates suboptimal results. However, the cross-validation of other species was performed satisfactorily. We can confidently deploy the proposed model, assuring its high-quality performance in identifying DNA methylation sites across different species, indicating that the proposed model has strong cross-validation performance.

### 4.4 The impact of the DNA vocabulary and label_words on model accuracy

To verify the algorithm’s effectiveness proposed in this article, the length of the DNA vocabulary library in the prompt template and the nucleotide length of the words in the label_words of the prompt verbalizer are changed to test their impact on the proposed model. In the following experiments, the nucleotide length in the DNA vocabulary refers to the length, encompassing all possible combinations of nucleotides ranging from 1, 2, … , up to that maximum length. For instance, if the nucleotide length is 6, then the DNA vocabulary includes nucleotide words that contain all combinations of nucleotides with lengths of 1, 2, 3, 4, 5, and 6.

#### 4.4.1 The impact of the number (length) of nucleotides in the DNA vocabulary library on the model

**FIGURE 6:**
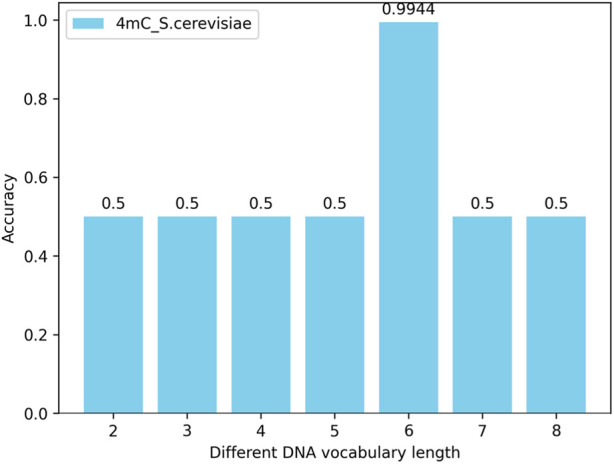
Impact of the number (length) of nucleotides in the DNA vocabulary library on the iDNA-OpenPrompt model.

By changing the length of the nucleotide vocabulary in the DNA vocabulary while keeping the nucleotide length of the words in the label_words of the prompt verbalizer at 6, tests are conducted on all species across three categories (4mC, 5hmC, 6mA) with the nucleotide numbers (lengths) of individual words in the DNA vocabulary library being 2, 3, 4, 5, 6, 7, and 8. The test results show that, with the nucleotide length of the words in the label_words of the prompt verbalizer unchanged, the highest model accuracy is achieved when the number of nucleotides of individual words in the DNA vocabulary is 6. Taking the 4mC species as an example, the model’s accuracy is illustrated in Figure 6.

#### 4.4.2 The impact of the number (length) of nucleotides in the label_words of the prompt verbalizer on the model

**FIGURE 7:**
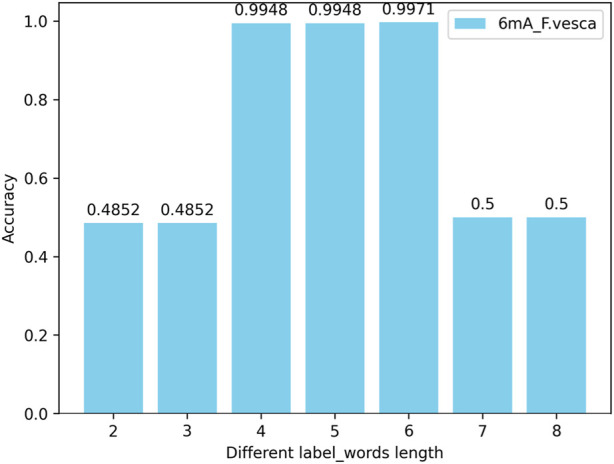
Accuracy of the number (length) of nucleotides in the label_words of the prompt verbalizer on the iDNA-OpenPrompt model.

In this experiment, by changing the length of the nucleotide vocabulary in the label_words of the prompt verbalizer while keeping the nucleotide length of the words in the DNA vocabulary of prompt template at 6, tests are conducted on all species across three categories (4mC, 5hmC, 6mA) with the nucleotide numbers (lengths) of individual words in the label_words being 2, 3, 4, 5, 6, 7, and 8. The test results indicate that, with the nucleotide length of the words in the DNA vocabulary of the prompt template unchanged, the highest model accuracy is achieved when the number of nucleotides of individual words in the label_words of the prompt marker is 6. Taking the 6mA_F.vesca species as an example, the model accuracy is illustrated in Figure 7.

#### 4.4.3 The accuracy of simultaneously changing the DNA vocabulary library and label_words of the iDNA-OpenPrompt model

**FIGURE 8:**
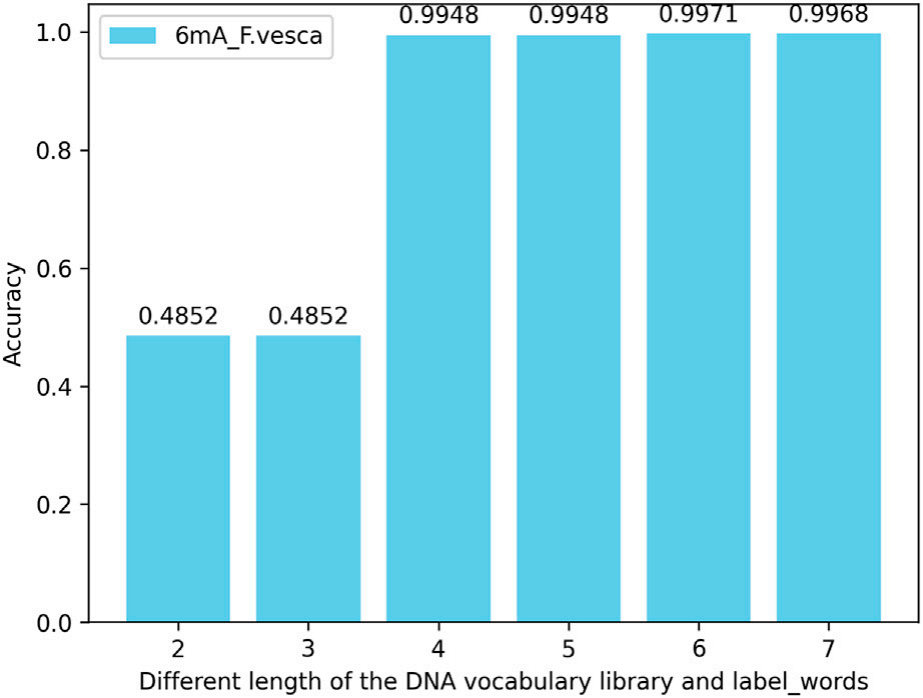
Accuracy of simultaneously changing the DNA vocabulary library and label_words of the iDNA-OpenPrompt model.

In this experiment, the extent of their impact on model performance is assessed by modifying the length of nucleotide vocabularies in both the DNA vocabulary of the prompt template and within the label_words of the prompt verbalizer. When the maximum length of nucleotide vocabularies in the DNA vocabulary and within the label_words is set to 2, 3, 4, 5, 6, and 7 for testing across multiple species within three methylation categories, the results reveal that the model’s accuracy peaked when both the maximum nucleotide vocabulary length in the DNA vocabulary and the nucleotide length within the label_words are 6. The performance does not improve further when the lengths are extended to 7, and the risk of overfitting the model increases when both lengths reach 8. Taking the 6mA species as an example, the model’s accuracy across various maximum lengths of nucleotide vocabularies in the DNA vocabulary and within the label_words of the prompt marker is illustrated in Figure 8.

## 5 Conclusion

The proposed iDNA-OpenPrompt model used the innovative OpenPrompt learning approach and combines a prompt template, prompt verbalizer, and PLM to construct the prompt learning framework. Moreover, a DNA vocabulary library, BERT tokenizer, and specific label words are also introduced into the model to enable accurate identification of DNA methylation sites. An extensive analysis is conducted to evaluate the model’s predictive capability, reliability, and consistency of the iDNA-OpenPrompt model. The experimental outcomes, covering 17 benchmark datasets that include various species and three distinct DNA methylation modifications, namely, 4mC, 5hmC, 6mA, consistently indicate that our model surpasses existing outstanding approaches regarding performance and robustness. The limitation to this model lies in that the DNA vocabulary in the prompt template is manually generated, and applying bioinformatics to other RNA sequences or other biological information sequences requires manual generation of their vocabularies anew. In future work, making vocabulary generation automatic and adaptable to other biological information sequences is one of the future research directions.

## Data Availability

Publicly available datasets were analyzed in this study. These data can be found at: https://github.com/Yyxx-1987/iDNA-OpenPrompt/tree/master/iDNA-OpenPrompt.

## References

[B1] Dai C. Jiang Y. Yin C. Su R. Zeng X. Zou Q. (2022). scIMC: a platform for benchmarking comparison and visualization analysis of scRNA-seq data imputation methods. Nucleic Acids Res. 50 (9), 4877–4899. 10.1093/nar/gkac317 35524568 PMC9122610

[B2] Devlin J. Chang M. -W. Lee K. Toutanova K. (2018). Bert: pre-training of deep bidirectional transformers for language understanding. Available at: https://arxiv.org/abs/1810.04805 . 10.48550/arXiv.1810.04805

[B3] Ding N. Hu S. Zhao W. Chen Y. Ding Z. Zheng H. -T. (2021). Openprompt: an open-source framework for prompt-learning. Available at: https://arxiv.org/abs/2111.01998 . 10.48550/arXiv.2111.01998

[B4] Duong N. TheAnh T. Le N. Q. K. DinhMinh P. YuYen O. (2021). An extensive examination of discovering 5-Methylcytosine Sites in Genome-Wide DNA Promoters using machine learning based approaches. IEEE/ACM Trans. Comput. Biol. Bioinforma. 10.1109/TCBB.2021.3082184 34014828

[B5] Haitao Y. Zhiming D. (2019). SNNRice6mA: a deep learning method for predicting DNA N6-methyladenine sites in rice genome. Front. Genet. 10, 1071. 10.3389/fgene.2019.01071 31681441 PMC6797597

[B6] Han X. Zhang Z. Ding N. Gu Y. Liu X. Huo Y. (2021). Pre-trained models: past, present and future. AI Open 2, 225–250. 10.1016/j.aiopen.2021.08.002

[B7] Han X. Zhao W. Ding N. Liu Z. Sun M. (2022). Ptr: prompt tuning with rules for text classification. AI Open 3, 182–192. 10.1016/j.aiopen.2022.11.003

[B8] Haodong X. Peilin J. Zhongming Z. (2020). Deep4mC: systematic assessment and computational prediction for DNA N4-methylcytosine sites by deep learning. Briefings Bioinforma. 22 (3). 10.1093/bib/bbaa099 PMC8138820 32578842

[B9] Hu S. Ding N. Wang H. Liu Z. Wang J. Li J. (2021). Knowledgeable prompt-tuning: incorporating knowledge into prompt verbalizer for text classification. Available at: https://arxiv.org/abs/2108.02035 . 10.48550/arXiv.2108.02035

[B10] iDNA-MS (2020). iDNA-MS web server.

[B11] Junru J. Yingying Y. Ruheng W. Xin Z. Chao P. Yi J. (2022). iDNA-ABF: multi-scale deep biological language learning model for the interpretable prediction of DNA methylations. Genome Biol. 23 (1), 219. 10.1186/s13059-022-02780-1 36253864 PMC9575223

[B12] Juntao C. Quan Z. Jing L. (2021). DeepM6ASeq-EL: prediction of human N6-methyladenosine (m6A) sites with LSTM and ensemble learning. Front. Comput. Sci. 16 (2), 162302. 10.1007/s11704-020-0180-0

[B13] Khanal J. Nazari I. Tayara H. Chong K. T. (2019). 4mCCNN: identification of N4-methylcytosine sites in prokaryotes using convolutional neural network. IEEE Access 7, 145455–145461. 10.1109/access.2019.2943169

[B14] Lester B. Al-Rfou R. Constant N. (2021). The power of scale for parameter-efficient prompt tuning. Available at: https://arxiv.org/abs/2104.08691 . 10.48550/arXiv.2104.08691

[B15] Leyi W. Shasha L. Eijy N. L. A. Ran S. Quan Z. (2019). Exploring sequence-based features for the improved prediction of DNA N4-methylcytosine sites in multiple species. Bioinforma. Oxf. Engl. 35 (8), 1326–1333. 10.1093/bioinformatics/bty824 30239627

[B16] Li F. Liu S. Li K. Zhang Y. Duan M. Yao Z. (2023a). EpiTEAmDNA: sequence feature representation via transfer learning and ensemble learning for identifying multiple DNA epigenetic modification types across species. Comput. Biol. Med. 160, 107030. 10.1016/j.compbiomed.2023.107030 37196456

[B17] Li Q. Cheng X. Song C. Liu T. (2023b). M6A-BERT-Stacking: a tissue-specific predictor for identifying RNA N6-methyladenosine sites based on BERT and stacking strategy. Symmetry 15 (3), 731. 10.3390/sym15030731

[B18] Li X. L. Liang P. (2021). Prefix-tuning: optimizing continuous prompts for generation. Available at: https://arxiv.org/abs/2101.00190 . 10.48550/arXiv.2101.00190

[B19] Liu X. Zheng Y. Du Z. Ding M. Qian Y. Yang Z. (2023). GPT understands, too. AI Open. 10.1016/j.aiopen.2023.08.012

[B20] Logan IV R. L. Balažević I. Wallace E. Petroni F. Singh S. Riedel S. (2021). Cutting down on prompts and parameters: simple few-shot learning with language models. Available at: https://arxiv.org/abs/2106.13353 . 10.48550/arXiv.2106.13353

[B21] Lv H. Dao F. -Y. Zhang D. Guan Z. -X. Yang H. Su W. (2020). iDNA-MS: an integrated computational tool for detecting DNA modification sites in multiple genomes. Iscience 23 (4), 100991. 10.1016/j.isci.2020.100991 32240948 PMC7115099

[B29] Maegawa S. Hinkal G. Kim H. S. Shen L. Zhang L. Zhang J. (2010). Widespread and tissue specific age-related DNA methylation changes in mice. Genome Res. 20 (3), 332–340. 10.1101/gr.096826.109 20107151 PMC2840983

[B22] Manavalan B. Basith S. Shin T. H. Wei L. Lee G. (2019). Meta-4mCpred: a sequence-based meta-predictor for accurate DNA 4mC site prediction using effective feature representation. Mol. Therapy-Nucleic Acids 16, 733–744. 10.1016/j.omtn.2019.04.019 PMC6540332 31146255

[B23] Mehedi H. M. Shaherin B. Shamima K. M. Gwang L. Balachandran M. Hiroyuki K. (2020). Meta-i6mA: an interspecies predictor for identifying DNA N6-methyladenine sites of plant genomes by exploiting informative features in an integrative machine-learning framework. Briefings Bioinforma. 22 (3). 10.1093/bib/bbaa202 32910169

[B24] Pian C. Zhang G. Li F. Fan X. (2020). MM-6mAPred: identifying DNA N6-methyladenine sites based on Markov model. Bioinformatics 36 (2), 388–392. 10.1093/bioinformatics/btz556 31297537

[B25] Quanzhong L. Jinxiang C. Yanze W. Shuqin L. Cangzhi J. Jiangning S. (2020). DeepTorrent: a deep learning-based approach for predicting DNA N4-methylcytosine sites. Briefings Bioinforma. 22 (3). 10.1093/bib/bbaa124 PMC8599298 32608476

[B26] Rao Z. Minghong L. (2020). Developing a multi-layer deep learning based predictive model to identify DNA N4-methylcytosine modifications. Front. Bioeng. Biotechnol. 8, 274. 10.3389/fbioe.2020.00274 32373597 PMC7186498

[B28] Schick T. Schütze H. (2020). Exploiting cloze questions for few shot text classification and natural language inference. Available at: https://arxiv.org/abs/2001.07676 . 10.48550/arXiv.2001.07676

[B30] Sho T. Mehedi H. M. HongWen D. Hiroyuki K. (2022). BERT6mA: prediction of DNA N6-methyladenine site using deep learning-based approaches. Briefings Bioinforma. 23 (2). 10.1093/bib/bbac053 PMC8921755 35225328

[B27] Ur R. M. Hilal T. Quan Z. To C. K. (2022). i6mA-Caps: a CapsuleNet-based framework for identifying DNA N6-methyladenine sites. Bioinformatics 38 (16), 3885–3891. 10.1093/bioinformatics/btac434 35771648

[B31] Wang R. Jiang Y. Jin J. Yin C. Yu H. Wang F. (2023). DeepBIO: an automated and interpretable deep-learning platform for high-throughput biological sequence prediction, functional annotation and visualization analysis. Nucleic Acids Res. 51 (7), 3017–3029. 10.1093/nar/gkad055 36796796 PMC10123094

[B32] Xia Y. Jia R. Yani C. Rao Z. Haixia L. Cuihua M. (2023). DRSN4mCPred: accurately predicting sites of DNA N4-methylcytosine using deep residual shrinkage network for diagnosis and treatment of gastrointestinal cancer in the precision medicine era. Front. Med. 10, 1187430. 10.3389/fmed.2023.1187430 PMC10192687 37215722

[B33] Xin C. Jun W. Qianyue L. Taigang L. (2021). BiLSTM-5mC: a bidirectional long short-term memory-based approach for predicting 5-methylcytosine sites in genome-wide DNA promoters. Molecules 26 (24), 7414. 10.3390/molecules26247414 34946497 PMC8704614

[B34] Yehudit B. Howard C. (2013). DNA methylation dynamics in health and disease. Nat. Struct. Mol. Biol. 20 (3), 274–281. 10.1038/nsmb.2518 23463312

[B35] Ying L. Yanan W. Zequn Z. Niannian L. Jun P. Jianjun T. (2022). Hyb4mC: a hybrid DNA2vec-based model for DNA N4-methylcytosine sites prediction. BMC Bioinforma. 23 (1), 258. 10.1186/s12859-022-04789-6 PMC9241225 35768759

[B36] Ying Z. Yan L. Jian X. Xiaoyu W. Xinxin P. Jiangning S. (2021). Leveraging the attention mechanism to improve the identification of DNA N6-methyladenine sites. Briefings Bioinforma. 22 (6), bbab351. 10.1093/bib/bbab351 PMC8575024 34459479

[B37] Yingying Y. Wenjia H. Junru J. Lizhen C. Rao Z. Leyi W. (2021). iDNA-ABT: advanced deep learning model for detecting DNA methylation with adaptive features and transductive information maximization. Bioinforma. Oxf. Engl. 37 (24), 4603–4610. 10.1093/bioinformatics/btab677 34601568

[B38] Zeng R. Liao M. (2021). 6mAPred-MSFF: a deep learning model for predicting DNA N6-methyladenine sites across species based on a multi-scale feature fusion mechanism. Appl. Sci. 11 (16), 7731. 10.3390/app11167731

[B39] Zhao Z. Wallace E. Feng S. Klein D. Singh S. (2021). “Calibrate before use: improving few-shot performance of language models,” in International Conference on Machine Learning PMLR, Maryland, USA, July 17-23, 2022. 10.3390/app11167731

[B40] Zhu Y. Wang Y. Qiang J. Wu X. (2023). Prompt-learning for short text classification. IEEE Trans. Knowl. Data Eng., 1–13. 10.1109/tkde.2023.3332787 36506788

[B41] Zou Q. Xing P. Wei L. Liu B. (2019). Gene2vec: gene subsequence embedding for prediction of mammalian N-6-methyladenosine sites from mRNA. Rna 25 (2), 205–218. 10.1261/rna.069112.118 30425123 PMC6348985

